# Use of routinely collected blood donation data for expanded HIV and Syphilis surveillance in Blantyre district, Malawi

**DOI:** 10.1371/journal.pone.0300647

**Published:** 2024-08-26

**Authors:** Emmanuel Singogo, Thomas Hartney, Sarah Bourdin, Maganizo Chagomerana, Evaristar Kudowa, Sydney Puerto-Meredith, Bridon M’baya, Godfrey Kadewere, Lucy Platt, Brian Rice, James R. Hargreaves, Sharon Weir, Mina C. Hosseinipour

**Affiliations:** 1 University of North Carolina Project-Lilongwe, Lilongwe, Malawi; 2 Faculty of Public Health and Policy, London School of Hygiene and Tropical Medicine, London, United Kingdom; 3 School of Medicine, University of North Carolina at Chapel Hill, Chapel Hill, Carolina, United States of America; 4 Malawi Blood Transfusion Services, Ministry of Health, Blantyre, Malawi; 5 Directorate of Health Technical Support Services, Ministry of Health, Lilongwe, Malawi; 6 School of Medicine and Population Health, University of Sheffield, Sheffield, United Kingdom; Mzuzu University Faculty of Information Science and Communications: Mzuzu University Faculty of Science Technology and Innovation, MALAWI

## Abstract

The World Health Organization recommends that all blood donations be screened for transfusion transmissible infections; these data are currently not incorporated into national disease surveillance efforts. We set out to use routinely collected data from blood donors in Blantyre district, Malawi to explore HIV and syphilis prevalence and identify sero-conversions among repeat donors. We conducted a retrospective cohort analysis of blood donation data collected by the Malawi Blood Transfusion Service from 2015 to 2021. All blood donations were routinely screened for HIV and syphilis. We characterized donor demographics as well as screening outcomes, including identifying sero-conversions among repeat donors who previously tested negative on their last donation. A total of 23,280 donations from 5,051 donors were recorded, with a median frequency of donations of 3 (IQR:2–6). Most donors were male (4,294; 85%) and students (3,262; 64.6%). Prevalence of HIV at first donation was 1.0% (52/5,051) and prevalence of syphilis was 1.6% (80/5,051); 52 HIV sero-conversions and 126 syphilis sero-conversions were identified, indicating an incidence rate per 1,000 person-years of 5.9 (95% CI: 4.7, 7.4) and 13.3 (95% CI:11.4, 15.4) respectively. Students had a lower prevalence of HIV and syphilis but higher risk of syphilis seroconversion. While blood donors are generally considered a low-risk population for HIV and syphilis, we were able to identify relatively high rates of undiagnosed HIV and syphilis infections among donors. Routinely collected data from national blood donation services may be used to better understand local HIV and syphilis epidemiology, with the potential to enhance disease surveillance systems. These findings may be used to identify priority prevention areas and populations in Blantyre district that can inform targeted interventions for improved disease prevention, testing and treatment.

## Introduction

Globally, HIV prevalence and incidence rates have decreased over time due to expanded HIV prevention programs and improved treatment therapies [1]. However, syphilis incidence and prevalence have been increasing among key populations globally [2]. In 2022, the World Health Organisation (WHO) launched global health sector strategies to end the epidemics of sexually transmitted infections, including HIV and syphilis by 2030 [3]. Countries with high HIV prevalence and incidence are implementing various strategies to improve HIV diagnosis and achieve the first 95 target of the Joint United Nations Programme on HIV/AIDS (UNAIDS) 2030 Fast-Track Targets. Some of these strategies include index testing, targeted screening at primary-level facilities, community testing, “moonlight" testing targeting key population in social venues, and adolescent programs such as DREAMS project [2,4]. To achieve the goal of reducing syphilis incidence by 90% by 2030, WHO strategies include screening priority populations for syphilis [3].

Malawi, like most countries in sub-Saharan Africa (SSA), relies on national HIV estimates from three main sources; the Spectrum model [5] which uses routinely collected HIV data; the Demographic and Health Surveys (DHS) [6] conducted every four years; and recently Population-based HIV Impact Assessments (PHIA) [7]. Data on syphilis prevalence among the general population is not reported in national population health surveys such as the DHS or Biological and Behavioural Surveillance Survey [7,8]. Instead, countries with limited resources, such as Malawi, rely heavily on syndromic diagnosis of genital ulcer disease to identify and treat syphilis cases. In order to have more reliable HIV and syphilis estimates, there is a need for expanded HIV surveillance in Malawi. A potential source for building up HIV and syphilis surveillance is routinely collected blood donation data. These blood donation data contain screening results for transfusion transmissible infections (TTIs) including HIV, syphilis and hepatitis B & C [9].

Since 2004, Malawi has an established national blood transfusion service and strategy for blood safety and availability and guidelines being implemented by the Malawi Blood Transfusion Services (MBTS) [10]. The strategy for blood safety and availability are in line with the WHO and International Federation of Red Cross and Red Crescent Societies recommendations [11,12]. Over the years, the MBTS has gradually expanded blood donation programs to cover all 28 districts in Malawi, except for Likoma Island due to logistical challenges. The majority of blood donors are voluntary non-remunerated blood donors (VNRBDs), with MBTS collecting blood from voluntary donors (70%) and some hospitals collect family replacement donors (30%) [13,14]. All blood donations collected by MBTS are routinely tested for TTIs including HIV and syphilis and test results data are consistently collected by MBTS and the Malawi Ministry of Health [15]. If well utilized, the TTI test results data can be used to expand HIV and syphilis surveillance beyond clinical and diagnostic services, and identify recent HIV and syphilis infections (sero-conversions) among repeat and regular donors.

Current program donation data show that typical Malawian blood donors tend to be young (median age below 25), in school (over 70%), and male (approximatively 80%) [9,16]. This is the population that is currently targeted by many HIV prevention strategies [2,4,7]. Even though an earlier study concluded that blood donor population is not a good proxy for a general population [17], we think these data are an underutilised source of information with great potential to contribute to disease surveillance and subsequent understanding of local variations in HIV disease burden over time. Therefore, in this analysis we aim to highlight the feasibility of using blood screening data for HIV and syphilis surveillance.

## Materials & methods

### Study design

This was a retrospective cohort study utilizing blood donation data collected by the Malawi Blood Transfusion Service (MBTS) from January 2015 to October 2021 in Blantyre district, Malawi. We only accessed the anonymized blood donation data on 10th January 2022. These blood donation data contain screening results for transfusion transmissible infections (TTIs) including HIV, syphilis and hepatitis B & C.

### Study settings

The MBTS routinely collects data through blood donation drives across 27 of the 28 districts in Malawi. In each administrative region, MBTS has a satellite office from which it operates to reach districts in the region: Blantyre (South), Balaka (East), Lilongwe (Central), and Mzuzu (North). While donation activities by MBTS target the whole population, the youth population in secondary schools constitute the majority of VNRBDs. MBTS teams conduct blood donation drives prior to donation visits at work places, schools, colleges villages, markets, and churches/mosques to encourage VNRBDs to donate blood and save lives. The requirement for blood donation in Malawi is to be aged between 16–65 years. Prior to donation, MBTS administers a pre-donation risk assessment questions that include having multiple sexual partners and recent engagement in risk behaviour to minimize donations from high risk individuals.

### Study population

Since 2004, the MBTS maintains an electronic database of uniquely identified donors and conducts regular quarterly blood bank hospital-level reporting to the Malawi Ministry of Health. The database has over 200,000 unique donors contributing over 350,000 donations. We used all data from a cumulative pool of 5,051 eligible unique blood donors collected from Blantyre district between 2015 and 2021. Data from all donations during this period were included in the analysis.

### Data collection and outcomes

Blood donation data were routinely collected by the MBTS through nationally harmonized MBTS data collection forms. Donors were classified into first-time and repeat donors using a unique donor ID that tracks individuals across multiple donations. Demographic variables were recorded, and included; sex at birth, marital status (categorized into married, single or divorced/widowed/ separated), occupation (categorized into student, employed, unemployed), and location of residence (at ward level for urban settings and traditional authority-level for rural settings). We calculated the number of unique donations for each donor over time between the first donation and the last donation. Both continuous and categorical ages (16–24, 25–34,35–44, 45+) in years at the time of the first donation were used. Location of residence at the ward-level rather than exact home address was used for spatial analysis. Ward names were matched to a separate GIS shapefile for Blantyre district.

HIV serology was done using Genscreen ULTRA HIV antigen-antibody enzyme immunoassay (EIA) reagents via the Evolis semi-automated platform. Supplementary testing with Determine antibody rapid test kits (Allere, Japan) was added in 2015 for those found positive with the EIA algorithm but later replaced in 2016 with chemiluminiscence immunoassays from Abbot (Germany) on the Architect i2000 platform. Syphilis serology was done using Bio-Rad (France) manual Treponema Pallidum haemagglutination assay (TPHA) reagents until the last quarter of 2016 and later replaced with chemiluminiscence immunoassays from Abbott (Germany) reagents detecting the same markers on the Architect i2000 platform. Both the algorithms for testing HIV and syphilis involved repeating in duplicate all initial positives and interpreting results based on the concordant two of the three.

### Outcomes and covariates

Primary outcomes included HIV and syphilis prevalence at the time of screening, and incidence of HIV and syphilis seroconversion for those with repeat donations. To examine HIV and syphilis seroconversion among repeated and regular blood donors, we included data from all donors who had successfully donated blood at least twice and had TTI results for these donations. The time point of seroconversion was defined as a mid-point between last negative test date and the positive test date. We assumed that there were no false test results and that all positive individuals were prevented from donating further so that all such individuals were negative for the TTI at least at the first donation. For each individual satisfying this requirement, the person-time at risk for that individual is the number of years between the first (negative) donation and either the last observed donation or the first donation with a positive TTI test, whichever comes first.

### Statistical analysis

Individual donor characteristics were summarised as counts and percentages for categorical variables, and medians (with interquartile ranges [IQR]) for continuous variables.

In univariable analyses, we used 95% confidence intervals to assess the strength of evidence for association between individual donor characteristics and donor status (first-time donors vs repeat-donors), and individual donor characteristics and HIV status at most recent donation, using either Fisher’s exact test or chi-squared as appropriate for categorical variables. Continuous variables (such as age) were compared using Wilcoxon rank-sum test.

We defined the overall incidence rate as the number of individuals who test positive at some donation divided by the sum of the person-time over all these individuals. These estimates were multiplied by 1,000 to provide incidence rate estimates per 1,000 person-years. Confidence intervals were constructed assuming the counts were distributed according to the Poisson distribution. Adjusted and unadjusted log-binomial regression models were used to the assess the association between: a) the covariates and HIV prevalence at first donation, b) the covariates and the syphilis prevalence at first donation, c) the covariates and the risk of HIV seroconversion among repeat donors, and d) the covariates and the risk of syphilis seroconversion among repeat donors. The adjusted models included the following variables: sex, age, occupation, and marital status.

We used crude prevalence rates for HIV and syphilis per traditional authority (TA) to produce TTI prevalence heat maps in order to highlight areas with disproportional burden of TTIs.

The analyses were performed using Stata software, version 14.1 (StataCorp LP, College Station,

Texas, USA) and spatial packages in R.

### Ethics approval and consent to participate

The MBTS obtained written informed consent to donate blood and use the data for research from all blood donors and/or their legal guardian(s). Prior to accessing the anonymized data, this analysis was approved by national ethics review board, the National Health Sciences Research Council (NHSRC (Protocol #20/07/2575). It was also approved by the Research Ethics Committee at London School of Hygiene & Tropical Medicine, United Kingdom. All research staff were trained on the study protocol, GCP, HSP and data collection standard operating procedures before being allowed to conduct any research activities.

## Results

A total of 23,280 donations from 5,051 donors were recorded, with 7 donors (0.1%) donating a maximum of 24 times and a median number of donations of 3 (IQR: 2–6) between 2015 and 2021. The majority of donors were single (83.9%), male (85%) and students (64.6%) at the time of their first donation (**Table 1**). Of the 5051 donors, 3837 (76%) donated blood at least twice. The median age of donors at first donation was 25 years (IQR: 20.6–31.8), with over half of donors aged 16–25 (54.1%). Those who donated multiple times were slightly older at first donation with median age being 25.4 (IQR: 21.2–31.9) compared to those who only donated once (23.7 [IQR: 19.3–31]). The distributions of age (categorical), sex, marital status, and occupation were similar between one-time donors and repeat donors.

**Table 1:** Descriptive characteristics of individual blood donors at most recent donation by donation status in Blantyre District (2015–2021).

	Total n (%)	One-time donors n (%)	Repeat donors (2+ times) n (%)
Total donors	5051(100)	1214 (24)	3837 (76)
Age (Yrs.)			
*Median (IQR)*	25(20.6, 31.8)	23.7(19.3, 31)	25.4(21.1, 31.9)
*16–25 yrs*.	2733(54.1)	720 (59.3)	2013 (52.5)
*26–35 yrs*.	1517(30.0)	316 (26.0)	1201 (31.3)
*36–45 yrs*.	561(11.1)	128 (10.5)	433 (11.3)
*45+ yrs*.	240(4.8)	50 (4.1)	190 (5.0)
Sex			
*Male*	4292(85.0)	939 (77.4)	3353 (87.4)
*Female*	759(15.0)	275(22.7)	484(12.6)
Marital Status			
*Single*	4238(83.9)	1067 (87.9)	3171 (82.6)
*Married*	784(15.5)	141 (11.6)	643 (16.8)
*Divorced/widowed*	29(0.6)	6 (0.5)	13 (0.6)
Occupation			
*Student*	3262(64.6)	837 (68.9)	2425 (63.2)
*Employed*	1433(28.4)	299 (24.6)	1134 (29.6)
*Unemployed*	54(1.1)	14 (1.2)	40 (1.0)
*Other*	302(6.0)	64 (5.3)	238 (6.2)

### HIV prevalence and incidence

Of the 5,051 donors screened for HIV, 126 (2.5%) tested positive, with 52 (41.3%) of these donors who tested positive for HIV were identified during first or baseline donation. The overall HIV prevalence at baseline was 1% (95% CI: 0.8, 1.4). Students had lower prevalence of HIV at baseline compared to other donors, which remained significant after adjusting for other variables, (adjusted prevalence ratio, aPR = 0.31; 95% CI: 0.15, 0.65) (**Table 2**). There were no significant differences in HIV prevalence observed among different sexes, marital statuses, and occupation.

**Table 2:** Characteristics of prevalent HIV and syphilis diagnosis on first donation among blood donors.

Characteristic		HIV				Syphilis			
		*Yes* *(N = 52)*	*No* *(N = 4999)*	*Unadjusted prevalence ratio* *(95% CI)*	*Adjusted prevalence ratio* *(95% CI)*	*Yes* *(N = 80)*	*No* *(N = 4971)*	*Unadjusted prevalence ratio* *(95% CI)*	*Adjusted prevalence ratio* *(95% CI)*
		*N (%)*	*N (%)*			*N (%)*	*N (%)*		
Sex									
	*Male*	43 (1.0)	4249 (99.0)	1.0	1.0	71 (1.7)	4221 (98.3)	1.0	1.0
	*Female*	9 (1.2)	750 (98.8)	1.18 (0.58, 2.42)	1.14 (0.56, 2.34)	9 (1.2)	750 (98.8)	0.72 (0.36, 1.43)	0.69 (0.35, 1.38)
Age									
	*16–25*	24 (0.8)	3133(99.2)	1.0	1.0	34 (1.1)	3123 (98.9)	1.0	1.0
	*26–35*	21 (1.7)	1233(98.3)	2.21 (1.23, 3.94)	1.49 (0.71, 3.11)	32 (2.6)	1222 (97.4)	2.37 (1.47, 3.82)	1.73 (0.97, 3.07)
	*36–45*	6 (1.3)	455(98.7)	1.71 (0.70, 4.17)	0.99 (0.33, 2.99)	13 (2.8)	448 (97.2)	2.62 (1.39, 4.92)	2.11 (0.93, 4.78)
	*≥46*	1 (0.6)	178 (99.4)	0.73 (0.10, 5.40)	0.39 (0.05, 3.31)	1 (0.6)	178 (99.4)	0.52 (0.07, 3.77)	0.39 (0.05, 3.09)
Marital status									
	*Single*	44 (1.0)	4194 (99.0)	1.0	1.0	70 (1.7)	4168 (98.3)	1.0	1.0
	*Married*	8 (1.0)	776 (99.0)	0.98 (0.46, 2.08)	0.70 (0.30, 1.63)	9 (1.2)	775 (98.8)	0.70 (0.35, 1.39)	0.46 (0.21, 0.98)
	*Divorced/widow*	0 (0.0)	29 (100.0)	-	-	1 (3.5)	28 (96.5)	2.09 (0.30, 14.52)	1.41 (0.21, 9.68)
Occupation									
	*Other*	27 (1.5)	1762 (98.5)	1.0	1.0	38 (2.1)	1751 (97.9)	1.0	1.0
	*Student*	25 (0.8)	3237 (99.2)	0.51 (0.30, 0.87)	0.31 (0.15, 0.65)	42 (1.3)	3220 (98.7)	0.61 (0.39, 0.94)	0.54 (0.30, 0.94)
Year									
	*2015*	2 (0.1)	2130 (99.9)	0.04 (0.01, 0.16)	0.03 (0.01, 0.13)	8 (0.4)	2124 (99.6)	0.13 (0.06, 0.28)	0.11 (0.05, 0.24)
	*2016*	4 (0.6)	706 (99.4)	0.24 (0.08, 0.67)	0.23 (0.08, 0.65)	6 (0.9)	704 (99.1)	0.30 (0.13, 0.70)	0.26 (0.12, 0.68)
	*2017*	31 (2.38)	1272 (97.6)	1.0	1.0	37 (2.8)	1266 (97.2)	1.0	1.0
	*2018*	9 (2.1)	425 (97.9)	0.87 (0.42, 1.82)	0.87 (0.42, 1.81)	7 (1.6)	427 (98.4)	0.57 (0.26, 1.26)	0.55 (0.25, 1.22)
	2019	2 (1.1)	189 (98.9)	0.44 (0.11, 1.82)	0.33 (0.08, 1.37)	7 (3.7)	184 (96.3)	1.29 (0.58, 2.85)	0.97 (0.43, 2.19)
	2020	2 (1.0)	194 (99.0)	0.43 (0.10, 1.78)	0.31 (0.07, 1.33)	8 (4.1)	188 (95.9)	1.44 (0.68, 3.04)	0.99 (0.46, 2.18)
	2021	2 (2.4)	83 (97.6)	0.99 (0.24, 4.06)	0.74 (0.18, 3.03)	7 (8.2)	78 (91.8)	2.90 (1.33, 6.31)	2.18 (0.99, 4.79)

Among repeat donors who previously tested negative, 80 HIV sero-conversions were identified over the study period, indicating an HIV incidence rate of 6.4 (95% CI: 5.1, 7.9) per 1,000 person-years (**Table 3**). HIV incidence rates did not significantly differ whether donors were students or not, nor by sex, age, and marital status.

**Table 3:** Incidence of HIV and syphilis by demographic characteristics.

Characteristic		New HIV Seroconversion					New Syphilis Seroconversion				
		Total patients N (%)	New cases	Person time (years)	Incidence per year (95%CI)	*p*-Value	Total patients (N = 3751) N (%)	New cases	Person time (years)	Incidence per year (95%CI)	*p*-Value
Overall		3833(100)	80(100)	12557.7	0.0064(0.0051, 0.0079)			171(100)	12483.4	0.0144 (0.0123, 0.0167)	
Sex											
	*Male*	3350(87.4)	75(93.8)	11396.9	0.0066(0.0052, 0.0082)	0.5	3348(87.4)	159 (93.0)		0.0140 (0.0119, 0.0164)	0.5
	*Female*	483(12.6)	5(6.2)	1160.8	0.0043(0.0014, 0.0101)		484(12.6)	12 (7.0)		0.0104 (0.0054, 0.0181)	
Age											
	*16–25*	2013(52.5)	38(47.5)	5177.7	0.0073(0.0052, 0.0101)	0.3	2016(52.6)	82(48.0)	5144.0	0.0159 (0.0127, 0.0198)	0.03
	*26–35*	1201(31.3)	29(36.3)	4821.7	0.0060(0.0040, 0.0086)		1195(31.2)	65(38.0)	4763.9	0.0136 (0.0105, 0.0174)	
	*36–45*	429(11.2)	11(13.8)	1792.9	0.0061(0.0031, 0.0110)		432(11.3)	18(10.5)	1816.7	0.0099 (0.0059, 0.0157)	
	*≥46*	190(5.0)	2(2.4)	765.4	0.0026(0.00073 0.0094)		189(4.9)	6(3.5)	758.8	0.0079 (0.0029, 0.0172)	
Marital status											
	*Single*	3168(82.7)	61(76.3)	9869.1	0.0062 (0.0047, 0.0079)	0.7	3168(82.7)	143 (83.6)	9803.0	0.0146(0.0123, 0.0172)	0.1
	*Married*	642(16.7)	19(23.7)	2594.1	0.0073(0.0044, 0.0114)		641(16.7)	28 (16.4)	2585.9	0.0108(0.0072, 0.0156)	
	*Divorced/* *Widowed*	23(0.6)	0 0.0)	94.5	0.0000 (0.0000, 0.0390)		23(0.6)	0(0.0)	94.5	0.0000(0.0000, 0.0390)	
Occupation											
	*Student*	2422(63.2)	42(52.5)	6926.6	0.0061(0.0044, 0.0082)	0.9	2422(63.2)	85 (49.7)	6885.0	0.0123 (0.0099, 0.0153)	0.3
	*Other*	1411(36.8)	38(47.5	5631.1	0.0067 (0.0048, 0.0093)		1410(36.8)	86(50.3)	5598.4	0.0154 (0.0123, 0.0190)	

### Syphilis prevalence and incidence

Of the 5,051 people screened for syphilis, 245 (4.9%) tested positive, with 80 (32.6%) testing positive at first donation. The prevalence of syphilis was significantly lower among students compared to other donors (aPR: 0.54; 95% CI:0.30, 0.94) (**Table 2)**. There were no statistically significant differences in syphilis prevalence among donors of different sexes and ages.

Among repeat donors who previously tested negative, 171 syphilis sero-conversions were identified over the study period, indicating a syphilis incidence rate of 14.4 (95% CI:12.3, 16.7) per 1,000 person-years (**Table 3**). Syphilis incidence significantly differed by age group (p = 0.03) only compared to other type of donors.

### Local geographical variations of prevalence of HIV and syphilis

**Fig 1:**
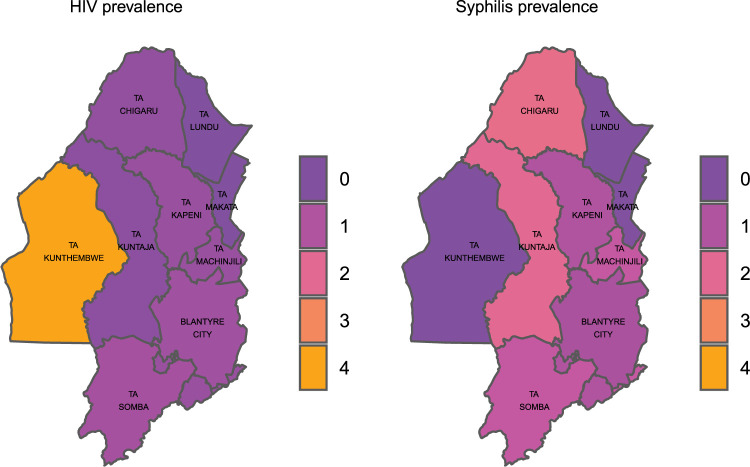
Prevalence of HIV and syphilis in Blantyre district by traditional authority.

The distribution of HIV and syphilis differed by traditional authority (**Fig 1**). HIV prevalence was highest in Kunthembwe (4.0%, 4/97), Somba (0.8%, 14/1783) and Blantyre city (0.7%, 161/23538). Syphilis prevalence was highest in Chigaru (2.2%, 30/1324), Kuntaja (2.0%, 23/1134) and Somba (1.3%, 24/1775). Prevalence for both HIV and syphilis was generally higher in TAs along the main M1 road (Somba, Blantyre City, Kapeni & Chigaru) than other TAs.

## Discussion

We present results from a retrospective cohort analysis of blood donation data collected by MBTS between October 2015 and May 2021 to estimate current HIV and syphilis prevalence and incidence and understand geographic distribution of HIV in Blantyre District. The majority of the 5,051 voluntary non-remunerated blood donors were single, male and students at the time of their first donation. There was a generally low HIV prevalence (2.1%) and a slightly high incidence of HIV seroconversion (6.4 per 1,000 person-years) among blood donors. Syphilis prevalence and incidence rate among the donors was 4.1% and 11.8 per 1,000 person-years, respectively. HIV and syphilis incidences were higher among men compared to women in both unadjusted and adjusted models. Students had a lower rate of HIV and syphilis prevalence and syphilis incidence even after adjustment for other characteristics. The distribution of HIV and syphilis differed by geographical location, with HIV prevalence in Kunthembwe being more than 4 times higher than any other TA within Blantyre district at 4.0% (4/97). Syphilis prevalence was slightly less heterogenous, with the highest prevalence being in Chigaru at 2.2% (30/1324) and lowest in Kunthembwe, Lundu and Makata at 0.0%. HIV and syphilis co-infection was low, only about 0.8% of donors were coinfected.

We observed an HIV prevalence among donors consistent with population-based prevalence estimates among men aged 15 to -34 years over the study period (1.3%-6.5%) [9,16,18–22]. While a lower prevalence than among the general population, this represents a substantial level of previously undiagnosed infections among people who can then be linked to confirmatory testing and treatment. Syphilis prevalence among the general population is not reported in national population health surveys except among key populations [8], but in this study we observed prevalence rates that are consistent with rates in other blood donation studies from other countries in the SSA region [20,21]. The HIV incidence we observed is higher than the incidence reported in recent population-based surveys (0.37% in 2015–2016 and 0.21% in 202–2021), but was close to the incidence observed among men aged 25–49 (0.40% - 0.49%) [7,22]. Notably we observed a higher incidence among men than women, which is the converse of the relationship seen in population-based surveys [7]. This suggests that donors may be a group at relatively high risk of seroconversion, however there is potential residual confounding which may warrant further study.

This dataset shares limitations common with other sources of routinely collected data. While rates of missing data were low, the number of covariates available was limited to age and employment status, meaning that there are likely to be confounders that cannot be accounted for in the analysis. Donors are unlikely to be representative of the general population, even among those who share similar demographic characteristics, and may be more likely to engage in healthcare-seeking behaviour or be subject to other forms of reporting or selection bias. Pre-donation screening assesses for HIV and syphilis risk and excludes those at high risk for these TTIs. Other issues that may limit the use of these data are reporting based on unconfirmed screening results, changes of test kits during this period which could have resulted in high potential for new screen false positives among negative blood donors.

This study demonstrates the feasibility of using routinely collected blood transfusion data to examine the prevalence of bloodborne infections among donors. The use of unique donor IDs means that this is a source of individual-level longitudinal data that allows for direct estimates of HIV and syphilis incidence without the need for modelling. The strengths of this data source include the combination of repeat testing and eligibility criteria meaning that these are likely to represent new infections rather than repeat diagnoses. In addition, the younger male population among donors are those who are typically less likely to test in clinical or community settings, especially for syphilis where Malawi does not currently have widespread levels of testing nationally. The use of geospatial analysis allows for identification of differential burden of infections across the Blantyre district and areas of especially high risk that may be used for focused prevention efforts.

This is a novel, low-cost, consistent and high-quality data source that has potential for future research in terms of supplementing existing surveillance systems and accessing a population less likely to test [23]. These populations will be more important going forward as the drive towards elimination of HIV as a public health concern means that these populations will represent the remaining pool of undiagnosed infections. While the estimates are specific to the donor population there are advantages of using these as a baseline level for examining trends over time and by geography and using this to identify changes in testing and incidence. Consideration should be given to systems that enable prompt linkage to care among those who are identified as newly infected. There is potential for other countries to take forward similar analyses. Additionally, we have only looked at MBTS data for HIV and syphilis within Blantyre, but this can be expanded to other TTIs on a national scale.

## Conclusion

Routinely collected data on TTIs in blood donors in Malawi is an underutilised source of information with great potential for reaching population groups less likely to test for HIV and syphilis in standard clinic setting. These data have potential to improve HIV and syphilis estimates nationally and understanding local variations in disease burden over time, both at individual level and aggregated level. The use of geospatial analysis is a useful tool for understanding the distribution of infections, identifying areas at especially high risk and exploring spatial relationships between risk factors and TTIs [24–27]. A better understanding of the local context can guide equitable resource allocation for effective prevention interventions.

## Supporting information

S1 File DATA- analysis dataset. (CSV)

## References

[1] UNAIDS. 2021 UNAIDS Global AIDS Update—Confronting inequalities—Lessons for pandemic responses from 40 years of AIDS. Geneva, Switzerland; 2021 Jul [cited 2022 Nov 29]. Available from: https://www.unaids.org/en/resources/documents/2021/2021-global-aids-update.

[2] USAID. DREAMS: Partnership to Reduce HIV/AIDS in Adolescent Girls and Young Women. 2022 [cited 2022 Nov 29]. Available from: https://www.usaid.gov/global-health/health-areas/hiv-and-aids/technical-areas/dreams.

[3] World Health Organization. Global health sector strategies 2022–2030. [cited 2024 Feb 14]. Available from: https://www.who.int/teams/global-hiv-hepatitis-and-stis-programmes/strategies/global-health-sector-strategies.

[4] UNAIDS. Understanding fast-Track: Accelerating Action to End the AIDS Epidemic by 20230. [cited 2024 Reb 14]. Available from https://www.unaids.org/sites/default/files/media_asset/201506_JC2743_Understanding_FastTrack_en.pdf.

[5] Stover J., Glaubius R., Kassanjee R. and Dugdale C.M. (2021), Updates to the Spectrum/AIM model for the UNAIDS 2020 HIV estimates. J Int AIDS Soc., 24: e25778. doi: 10.1002/jia2.25778 34546648 PMC8454674

[6] National Statistical Office (NSO) Malawi, ICF. Malawi Demographic and Health Survey 2015–16. Zomba, Malawi and Rockville, Maryland, USA: NSO and ICF; 2017 Feb [cited 2021 Jul 7]. Available from: https://dhsprogram.com/pubs/pdf/FR319/FR319.pdf.

[7] Ministry of Health (MOH) Malawi. Malawi Population-Based HIV Impact Assessment (MPHIA) 2015–2016: Final Report. Lilongwe, Malawi: Ministry of Health, Malawi; 2018 Oct [cited 2021 Jul 7]. Available from: https://phia.icap.columbia.edu/wp-content/uploads/2019/08/MPHIA-Final-Report_web.pdf.

[8] National Statistical Office. Malawi Biological and Behavioural Surveillance Survey (BBSS) - 2019–2020. Final report. Zomba, Malawi, 2020[cited 2024 Feb 14]. Available from: http://www.nsomalawi.mw/images/2019-2020_Malawi_BBSS_Report_FINAL.pdf.

[9] M’baya B, Jumbe V, Samuel V, M’bwana R, Mangani C. Seroprevalence and trends in transfusion transmissible infections among voluntary non-remunerated blood donors at the Malawi Blood Transfusion Service-a time trend study. Malawi Med J J Med Assoc Malawi. 2019 Jun;31(2):118–25. doi: 10.4314/mmj.v31i2.3 31452844 PMC6698631

[10] Malawi Blood Transfusion Services. 2021 [cited 2021 Jul 8]. Available from: http://mbtsmalawi.com/.

[11] World Health Organization. Blood safety and availability. 2023 June 1 [cited 2024 Feb 14]. Available from: https://www.who.int/news-room/fact-sheets/detail/blood-safety-and-availability.

[12] International Federation of Red Cross and Red Crescent Societies. Promoting safe and sustainable national blood systems policy. 2022 Jun 21 [cited 2024 Feb 14]. Available from: https://www.ifrc.org/document/promoting-safe-and-sustainable-national-blood-systems-policy.

[13] Njolomole SE, M’baya B, Ndhlovu D, M’bwana R, Samuel V, Jumbe Vet al. Post Baseline Situational Analysis of Blood Safety in Malawi 2015. Blantyre. 2017[cited 2024 Feb 14]. Available from: https://mbtsmalawi.com/wp-content/uploads/2022/10/Post-Baseline-Blood-safety-Situational-Analysis-Report-with-all-annexes-2.pdf.

[14] Kongnyuy EJ, Broek N van den. Availability and safety of blood for transfusion in three districts in Malawi. Trop Med Health. 2008;36(4):155–62.

[15] Malawi Ministry of Health. Integrated HIV Program Report Q4; Lilongwe, Malawi. 2019 https://dms.hiv.health.gov.mw/dataset/malawi-integrated-hiv-program-repo.

[16] Singogo E, Chagomerana M, Van Ryn C, M’bwana R, Likaka A, M’baya B, et al. Prevalence and incidence of transfusion-transmissible infections among blood donors in Malawi: A population-level study. Transfus Med. 2023 Dec;33(6):483–496. doi: 10.1111/tme.13006 37828838 PMC11096640

[17] Vermeulen M, Swanevelder R, Chowdhury D, et al. Use of blood donor screening to monitor prevalence of HIV and hepatitis B and C viruses. South Africa Emerg Infect Dis. 2017; 23(9): 1560–1563. 28820374 10.3201/eid2309.161594 PMC5572879

[18] Birhaneselassie M. Prevalence of Transfusion-Transmissible Infections in Donors to an Ethiopian Blood Bank Between 2009 and 2013 and Donation Factors That Would Improve the Safety of the Blood Supply in Underdeveloped Countries. Lab Med. 2016 May;47(2):134–9. doi: 10.1093/labmed/lmw003 27069031

[19] Mohamed Z, Kim JU, Magesa A, Kasubi M, Feldman SF, Chevaliez S, et al. High prevalence and poor linkage to care of transfusion-transmitted infections among blood donors in Dar-es-Salaam, Tanzania. J Viral Hepat. 2019;26(6):750–6. doi: 10.1111/jvh.13073 30712273 PMC6563112

[20] Bartonjo G. Prevalence and factors associated with transfusion transmissible infections among blood donors at Regional blood transfusion center Nakuru and Tenwek Mission Hospital, Kenya [Internet] [Thesis]. Laboratory Management and Epidemiology, JKUAT; 2013 [cited 2022 Nov 28]. Available from: http://localhost/xmlui/handle/123456789/1902.

[21] Okoroiwu HU, Okafor IM, Asemota EA, Okpokam DC. Seroprevalence of transfusion-transmissible infections (HBV, HCV, syphilis and HIV) among prospective blood donors in a tertiary health care facility in Calabar, Nigeria; an eleven years evaluation. BMC Public Health. 2018 May 22;18(1):645. doi: 10.1186/s12889-018-5555-x 29788937 PMC5964952

[22] Ministry of Health M. Malawi Population-Based HIV Impact Assessment (MPHIA) 2020–2021: Summary Sheet [Internet]. Lilongwe, Malawi: Ministry of Health, Malawi; 2022 Mar [cited 2022 Nov 28]. Available from: https://phia.icap.columbia.edu/malawi-summary-sheet-2/.

[23] Foster V, Young A. The use of routinely collected patient data for research: A critical review. Health (N Y). 2012 Jul 1;16(4):448–63. doi: 10.1177/1363459311425513 22071234

[24] Qin Q, Guo W, Tang W, Mahapatra T, Wang L, Zhang N, et al. Spatial Analysis of the Human Immunodeficiency Virus Epidemic among Men Who Have Sex with Men in China, 2006–2015. Clin Infect Dis. 2017 Apr 1;64(7):956–63. doi: 10.1093/cid/cix031 28362948 PMC5439342

[25] Bautista CT, Sateren WB, Sanchez JL, Singer DE, Scott P. Geographic mapping of HIV infection among civilian applicants for United States military service. Health Place. 2008 Sep;14(3):608–15. doi: 10.1016/j.healthplace.2007.10.004 18024132

[26] Hixson BA, Omer SB, del Rio C, Frew PM. Spatial clustering of HIV prevalence in Atlanta, Georgia and population characteristics associated with case concentrations. J Urban Health Bull N Y Acad Med. 2011 Feb;88(1):129–41. doi: 10.1007/s11524-010-9510-0 21249526 PMC3042078

[27] Wabiri N, Shisana O, Zuma K, Freeman J. Assessing the spatial nonstationarity in relationship between local patterns of HIV infections and the covariates in South Africa: A geographically weighted regression analysis. Spat Spatio-Temporal Epidemiol. 2016 Feb;16:88–99. doi: 10.1016/j.sste.2015.12.003 26919758

